# Current treatment and outcomes of traumatic sternovertebral fractures: a systematic review

**DOI:** 10.1007/s00068-020-01505-y

**Published:** 2020-10-01

**Authors:** Dorine S. Klei, F. Cumhur Öner, Luke P. H. Leenen, Karlijn J. P. van Wessem

**Affiliations:** 1 Department of Trauma Surgery, Medical Centre Utrecht, University, Suite G04.232, Heidelberglaan 100, Utrecht, 3584 CX The Netherlands; 2 Orthopaedic Surgery, Department of Orthopaedic Surgery, University Medical Centre Utrecht, Utrecht, The Netherlands

**Keywords:** Traumatic sternal and spinal fractures, Sternovertebral fractures, Treatment, Outcomes, Systematic review

## Abstract

**Purpose:**

Combined sternal and spinal fractures are rare traumatic injuries with significant risk of spinal and thoracic wall instability. Controversy remains with regard to treatment strategies and the biomechanical need for sternal fixation to achieve spinal healing. The present study aimed to assess outcomes of sternovertebral fracture treatment.

**Methods:**

A systematic review of literature on the treatment of traumatic sternovertebral fractures was conducted. Original studies published after 1990, reporting sternal and spinal healing or stability were included. Studies not reporting treatment outcomes were excluded.

**Results:**

Six studies were included in this review, with a total study population of 98 patients: 2 case series, 3 case reports, and 1 retrospective cohort study. 10 per cent of sternal fractures showed displacement. Most spinal fractures were located in the thoracic spine and were AOSpine type A (51%), type B (35%), or type C (14%). 14 per cent of sternal fractures and 49% of spinal fractures were surgically treated. Sternal treatment failure occurred in 5% of patients and biomechanical spinal failure in 8%. There were no differences in treatment failure between conservative and operative treatment.

**Conclusion:**

Literature on traumatic sternovertebral fracture treatment is sparse. Findings indicate that in most patients, sternal fixation is not required to achieve sternal and spinal stability. However, results of the current review should be cautiously interpreted, since most included studies were of poor quality.

**Electronic supplementary material:**

The online version of this article (10.1007/s00068-020-01505-y) contains supplementary material, which is available to authorized users.

## Background

Combined sternal and spinal fractures, also known as ‘sternovertebral’ fractures, are rare injuries with an estimated incidence of 0.64% in traffic victims [1] and 1% in trauma patients admitted to a level-1 trauma centre [2]. These injuries are primarily caused by a combination of direct impact and indirect flexion-compression or flexion-rotation forces, due to high deceleration in motor vehicle accidents [1, 3–7]. In literature, the thoracic spine is regarded as the preferential location for sternovertebral fractures [1, 4, 7–12]. However, concomitant cervical or lumbar spinal fractures with an incidence similar to or higher than thoracic spinal fractures are also reported [1, 2, 4–6, 13–20]. Associated injuries markedly increase morbidity and mortality [1, 2, 5–7, 12–14, 18, 19, 21, 22].

The relationship between spinal, sternal, and rib fractures is well-established. According to the four-column spine model, the thoracic cage, composed of sternum and ribs, acts as the crucial fourth column of mechanical support and stability for the thoracic spine [8, 23]. Sternovertebral fractures might therefore severely impair spinal and thoracic wall instability.

Few studies have addressed treatment methods and outcomes of sternovertebral fractures, and most had a small patient population; a retrospective cohort study conducted at our level-1 trauma centre is the largest study to date [2]. Uniform treatment strategies are lacking. Most sternal fractures are safely managed conservatively, but surgery might be indicated in case of (secondary) dislocation (≥ 1 sternal width), sternal instability or deformity, severe pain leading to respiratory insufficiency, and fracture non-union [24–26]. Indications for spinal fixation are unstable fractures and fractures with associated spinal cord injury [27, 28]. However, in case of combined sternovertebral fractures, sternal fixation might biomechanically be crucial to achieve sternal and spinal fracture healing. Some authors argued that treatment depends on spinal fracture level [1] or that fixation of both sternal and spinal fractures is imperative for adequate spinal support [26]. Others postulated that spinal fixation alone is sufficient [2, 10].

In short, standardised treatment recommendations for sternovertebral fractures are lacking. The aim of the present study was to conduct a systematic review of literature and provide an overview of the outcomes of sternovertebral fracture treatment.

## Methods

Academic search engines PubMed and Embase/MEDLINE were searched with the terms ‘sternum’, ‘spine’, ‘fracture’, ‘dislocation’, ‘injury’, ‘treatment’, and their respective synonyms, both as free entry terms and Mesh (PubMed) and Emtree (Embase/MEDLINE) terms (online Appendix A). The term ‘dislocation’ was added to our search terms to expand the scope of our literature search, since sternal and spinal dislocations generally concern fracture-dislocations. No filters or language restrictions were applied to the literature search.

Primary and secondary outcome parameters were formulated for the assessment of included articles (Table 1). Original studies published after 1990, consisting of adult patients with combined traumatic sternovertebral fractures, reporting at least one primary outcome parameter for both the sternal and spinal fractures, were included in this review. Studies that did not fulfil these inclusion criteria, studies for which no full-text was available, studies focusing on sternal fractures due to cardiopulmonary resuscitation, and review studies were excluded. Due to the limited available research, all study types were eligible for inclusion. Included studies were assessed for cross-references.

**Table 1:** Parameters for the assessment of included articles

Study characteristics		Publication year
		Journal
		Country
		Study type
		Study period
		Number of included patients
		Length of follow-up
Patient characteristics	General	Age
		Gender
		Injury mechanism
		Associated injuries
		Comorbidities
	Sternal injury	Type of injury
		Location
		Dislocation
	Spinal injury	Location
		AOSpine-classification (A / B / C)
		Neurological deficit
Treatment methods		Primary treatment (surgical or conservative)
		Conservative treatment method (if applicable)
Treatment outcomes	Primary outcome parameters	Sternal treatment failure
		Spinal treatment failure
	Secondary outcome parameters	ICU admission
		Pneumonia
		Wound infection
		Hospital length of stay

*MS-joint* manubriosternal joint, *ICU* intensive care unit

The quality of included studies was assessed using the MINORS quality assessment criteria, a validated methodological index for non-randomised studies [29]. In the MINORS-assessment, a score of 2 points is awarded for a reported and adequate criterion, 1 point for a reported but inadequate criterion, and 0 points for a non-reported criterion. For case series and case reports, only the eight criteria for non-comparative studies were used (with a maximum score of 16 points). For the retrospective cohort study, the four criteria for comparative studies were added (with a maximum score of 24 points). For quality assessment, appropriate endpoints for treatment outcome were defined as reported fracture healing (confirmed by radiographic analysis or directly seen at re-operation), report of a clinically healed fracture, or reported increase in spinal angulation or loss of vertebral height; primary outcome parameters had to be reported for all included patients. An appropriate follow-up period was defined as ≥ 3 months.

Finally, the parameters in Table 1 were extracted from the included studies. Treatment failure was defined as reoperation (after primary surgical treatment), operation secondary after conservative treatment, a surgical indication due to secondary dislocation or non-union, or unreported treatment outcomes. For spinal fractures, a distinction was made between technical treatment failure (malpositioned screws or pain because of osteosynthesis materials) and biomechanical failure. Unstable fractures were defined as AOSpine type B or type C fractures. Two authors (DK and KW) independently performed the literature search and quality assessment. In the event of disagreement, joint re-assessment of the relevant study resulted in final consensus.

### Statistical analysis

Statistical analysis was performed using R Statistics (an open-source integrated development environment for statistical computing). Subgroup analysis was carried out for the four different treatment groups. Normality of continuous variables was assessed through Kernel density scores. For normal distributions, values were calculated as mean (range); for non-normal distributions, outcomes were expressed as median [interquartile range, IQR]; significant differences were determined with a one-way ANOVA or Kruskal–Wallis Rank Sum Test, respectively. For categorical variables, outcomes were calculated as number (percentage); because of small group sizes, significant differences were identified using Fisher’s exact test. A two-sided *P* value of < 0.05 was considered statistically significant.

## Results

### Search results

**Fig. 1:**
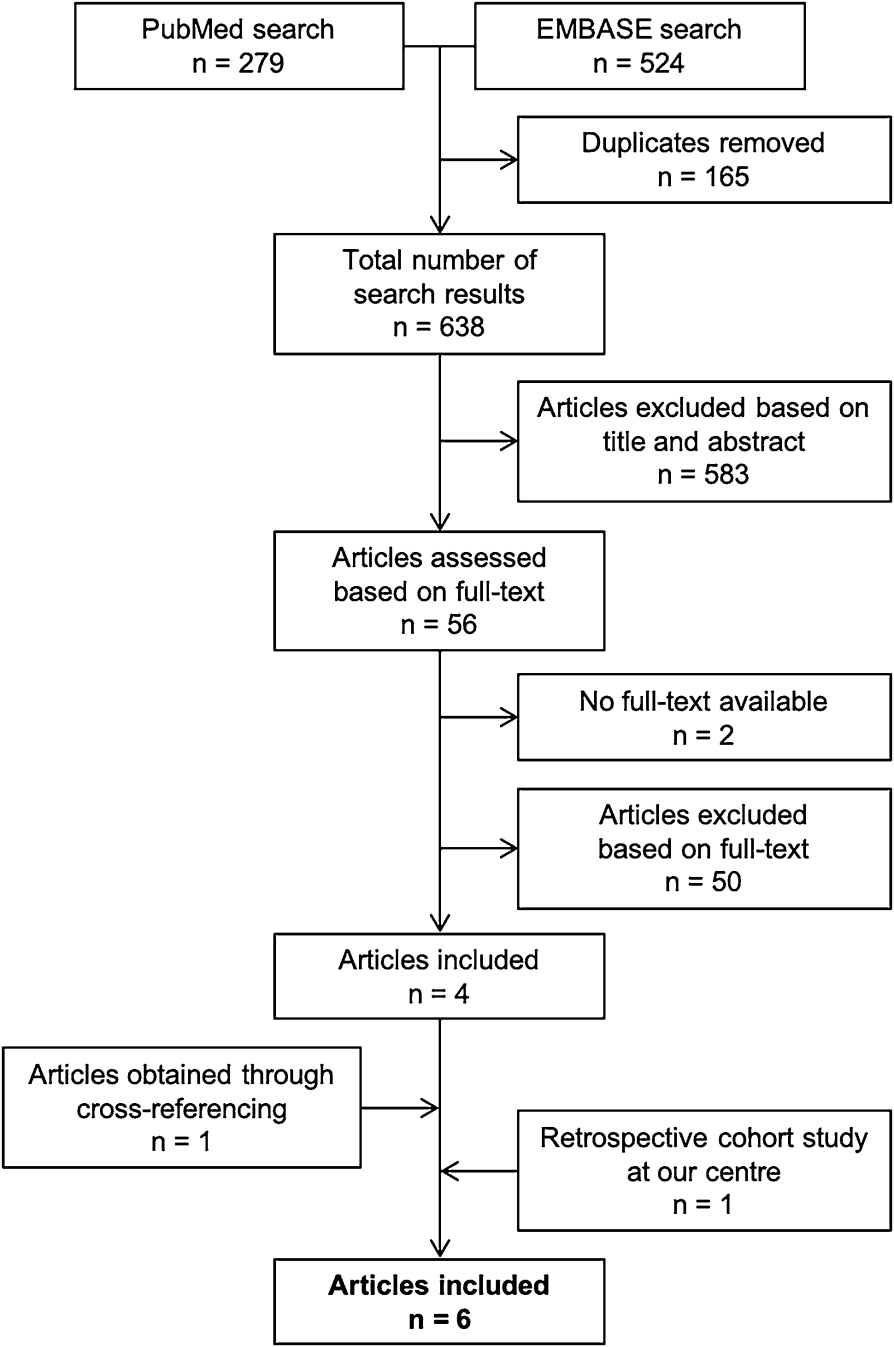
Search summary

The literature search was performed on 22 February 2020. The PubMed search resulted in 279 hits and the Embase/MEDLINE search yielded 524 hits, resulting in a total of 803 hits. After removal of 165 duplicates, 638 studies were assessed based on title and abstract. 583 articles did not fulfil the inclusion criteria and were therefore excluded. 56 studies were assessed based on full-text, of which 4 studies were included in the current review. For two studies, no full-text article was available and these studies were therefore excluded. Cross-referencing yielded one additional included study. The results of a retrospective cohort study conducted at our level-1 trauma centre, which appeared online in February 2020, were also included in the analysis (Fig. 1).

### Quality assessment

For case reports and case series, the total quality score ranged from 3 to 6 out of 16 points. The retrospective cohort study scored 17 out of 24 quality points. Several criteria, such as prospective data collection, unbiased measurement of study end points, and prospective calculation of study size, were not reported by any study (Table 2).

**Table 2:** MINORS quality assessment

Study	Clearly stated aim	Inclusion consecutive patients	Prospective data collection	Appropriate endpoints to the aim	Unbiased Study endpoint	Appropriate follow-up period	Loss to follow-up < 5%	Prospective calculation of study size	Adequate control group	Contemporary groups	Baseline equivalence of groups	Adequate statistical analysis	Total quality score
Jiang et al. [11]	1	N/a	0	2	0	2	N/a	N/a	N/a	N/a	N/a	N/a	**5 / 16**
Klei et al. [24]	2	2	0	2	0	2	2	0	2	2	1	2	**17 / 24**
Krinner et al. [3]	1	0	0	1	0	2	2	0	N/a	N/a	N/a	N/a	**6 / 16**
Labbe et al. [10]	1	0	0	2	0	0	0	0	N/a	N/a	N/a	N/a	**3 / 16**
Regauer et al. [26]	1	N/a	0	2	0	2	N/a	N/a	N/a	N/a	N/a	N/a	**5 / 16**
Sarkeshik et al. [33]	1	N/a	0	1	0	2	N/a	N/a	N/a	N/a	N/a	N/a	**4 / 16**

MINORS criteria: 0 = not reported, 1 = reported but inadequate, 2 = reported and adequate. N/a, not applicable

For case reports and case series, the eight criteria for non-comparative studies were used (maximum total score of 16 points). For the retrospective cohort study, four criteria for comperative studies were added (maximum total score of 24 points)

### Study characteristics

Studies were published from 2009 to 2020. Two studies were case series, three were case reports, and one was a retrospective cohort study. Study periods ranged from 3 to 10 years, with a total study period of 16 years and 5 months. Together, the studies comprised 119 patients. Treatment outcomes were reported for 98 patients; these patients were included in our analysis. Follow-up length ranged from 6 to 32 months, but was not reported by Labbe [10] (Table 3).

**Table 3:** Characteristics of included studies

Study	Study type		Study period	Number of patients		Follow-up length
Jiang et al. [11]	Case report		–	1		14 months
Klei et al. [24]	Retrospective cohort		10 years	73		13 (6–22) months
Krinner et al. [3]	Case series		3 years and 5 months	11		–
Labbe et al. [10]	Case series		3 years	32 (treatment outcomes for 11)		24 months
Regauer et al. [26]	Case report		–	1		32 months
Sarkeshik et al. [33]	Case report		–	1		12 months
Total	Case report	(*n* = 3)	16 years and 5 months	Total number of patients	119	Range 6 – 34 months
	Case series	(*n* = 2)		Included in analysis	98	
	Retrospective cohort	(*n* = 1)				

–, unknown

### Patient characteristics

98 patients were included in analysis. There were four treatment combinations: conservative treatment for both sternum and spine (SternumCONS/SpineCONS, *n* = 43), conservative treatment for sternum and operative treatment for spine (SternumCONS/SpineOP, *n* = 41), operative treatment for sternum and conservative treatment for spine (SternumOP/SpineCONS, *n* = 7), and operative treatment for both sternum and spine (SternumOP/SpineOP, *n* = 7) (Table 4).

**Table 4:** Patient characteristics^a^

	Overall *n* = 98		Sternum CONS Spine CONS *n* = 43		Sternum CONS Spine OP *n* = 41		Sternum OP Spine CONS *n* = 7		Sternum OP Spine OP *n* = 7		*p* value
Age in years, mean (range)	49	(16–93)	52	(24–93)	43	(16–76)	52	(27–73)	57	(19–85)	0.044*
Gender (male)	69	(70)	34	(79)	28	(68)	3	(43)	3	(43)	0.164
Mechanism of injury, *n* (%)											0.013*
Fall ≤ 3 m	13	(13)	1	(2)	8	(20)	2	(29)	2	(29)	
Fall > 3 m	19	(19)	7	(16)	10	(24)	0		2	(29)	
Traffic	62	(63)	34	(79)	21	(51)	5	(71)	2	(29)	
Other	4	(4)	1	(2)	2	(5)	0		1	(14)	
Sternal fracture characteristics											
Injury type, *n* (%)											0.561
Fracture	97	(99)	43	(100)	40	(98)	7	(100)	7	(100)	
Subluxation	1	(1)	0		1	(2)	0		0		
Number of sternal fractures, *n* (%)											0.099
1 fracture	83	(85)	40	(93)	33	(81)	5	(71)	5	(71)	
2 fractures	15	(15)	3	(7)	8	(20)	2	(29)	2	(29)	
Sternal fracture location, *n* (%) ^b^											
Manubrium	42	(43)	24	(56)	13	(32)	3	(43)	2	(29)	0.138
MS-joint	16	(16)	1	(2)	11	(27)	1	(14)	3	(43)	0.001*
Sternal body	54	(55)	20	(47)	25	(61)	5	(71)	4	(57)	0.485
Xiphoid process	1	(1)	1	(2)	0		0		0		1.000
Dislocation of sternal fracture, *n* (%)	10	(10)	1	(2)	6	(15)	0		3	(43)	0.010*
Spinal fracture characteristics											
AOSpine classification, *n* (%)											< 0.001*
A	50	(51)	36	(84)	6	(15)	7	(100)	1	(14)	
B	34	(35)	6	(14)	25	(61)	0		3	(43)	
C	14	(14)	1	(2)	10	(24)	0		3	(43)	
Spinal fracture location, *n* (%) ^c^											
Upper cervical (C0-C2) ^d^	2	(2)	1	(2)	0		1	(14)	0		0.267
Cervical subaxial (C3-C7)	15	(15)	12	(28)	3	(7)	0		0		0.037*
Upper thoracic (T1-T4)	22	(22)	8	(19)	9	(22)	3	(43)	2	(29)	0.437
Thoracic (T5-T9)	28	(29)	7	(16)	15	(37)	3	(43)	3	(43)	0.083
Thoracolumbar (T10-L2)	37	(38)	19	(44)	15	(37)	1	(14)	2	(29)	0.485
Lower lumbar (L3-L5) ^d^	2	(2)	2	(5)	0		0		0		0.629
Neurological status, *n* (%)											< 0.001*
Neurological deficit (N1–N4)	21	(21)	1	(2)	17	(42)	0		3	(43)	
Unknown (NX)	2	(2)	1	(2)	1	(2)	0		0		
Associated injuries											
Associated thoracic injuries, *n* (%)	82	(83)	36	(84)	34	(83)	6	(86)	6	(86)	1.000
Rib fracture	70	(71)	30	(70)	30	(73)	5	(71)	5	(71)	0.979
Clavicular fracture	19	(19)	8	(19)	7	(17)	2	(29)	2	(29)	0.768
Lung contusion	39	(40)	24	(56)	13	(32)	1	(14)	1	(14)	0.022*
Pneumothorax	40	(41)	19	(44)	18	(44)	2	(29)	1	(14)	0.479
Haemothorax	25	(26)	9	(21)	13	(32)	1	(14)	2	(29)	0.628
Cardiac contusion	7	(7)	3	(7)	4	(10)	0		0		0.905
Other thoracic injuries	23	(24)	10	(23)	8	(20)	2	(29)	3	(43)	0.581
Other associated injuries, *n* (%)	50	(51)	24	(56)	22	(54)	2	(29)	2	(29)	0.374
Cerebral injury	18	(18)	7	(16)	10	(24)	0		1	(14)	0.519
Abdominal injury	22	(22)	14	(33)	8	(20)	0		0		0.099
Extremity injury	41	(42)	19	(44)	18	(44)	2	(29)	2	(29)	0.793
FU in months, median [IQR]	17	(8–24)	12	(5–25)	17	(8–23)	24	(17–24)	24	(24–24)	0.072

*CONS* conservative treatment, *OP* operative treatment, *MS-joint* manubriosternal joint, *IQR* interquartile range, *FU* follow-up duration

^*^Statistically significant difference (*p* < 0.05)

^a^Due to rounding off, percentages might not add up to 100%

^b^Sternal fracture location is displayed as the percentage of patients with a sternal fracture in a particular location. 15 patients had two sternal fractures and were counted in two groups

^c^Spinal fracture location is displayed as the percentage of patients with a spinal fracture in a particular location. 8 patients had spinal fractures of similar severity in multiple spinal levels and were counted in two groups

^d^Patients with only upper cervical or lower lumbar spinal fractures were not included in any study. However, four patients had upper cervical or lower lumbar fractures in combination with other spinal fractures

Patients in the SternumCONS/SpineOP group were younger and patients in the SternumOP/SpineOP group were older than patients in the other treatment groups (*p* = 0.044). Many patients (79%) in the SternumCONS/SpineCONS group had a traffic accident, while only one patient in this group fell from ≤ 3 m height (*p* = 0.013).

In the SternumOP/SpineOP group, significantly more patients had a manubrial fracture and/or a sternal fracture dislocation (*p* = 0.001 and *p* = 0.010, respectively). In the SternumCONS/SpineCONS group, more patients had a type A spinal fracture, while in the SternumCONS/SpineOP group, more patients had a type B fracture (*p* < 0.001). Patients in the SternumCONS/SpineCONS group were more likely to have a subaxial spinal fracture (*p* = 0.037). Patients with isolated upper cervical or lower lumbar spinal fractures were not included in any study; however, four patients had upper cervical or lower lumbar fractures in combination with other spinal fractures and were thus included in our analysis.

In the SternumCONS/SpineOP group, more patients had a neurological spinal deficit, in contrast to patients in the conservative spinal treatment groups where only one patient had spinal cord injury (*p* < 0.001). Patients in the SternumCONS/SpineCONS group were more likely to have a pulmonary contusion compared to patients in other treatment groups (*p* = 0.022).

There were no statistical differences in sternal injury type, number of sternal fractures, associated other injuries, or follow-up duration between the treatment groups. Most studies did not provide information on pre-existent comorbidities and ICU admission; these parameters were excluded from analysis (Table 4).

### Treatment methods

In 84 patients (86%), the sternal fracture was conservatively treated. 14 patients (14%) underwent sternal fixation: indications were persistent severe chest discomfort (*n* = 1), a combination of respiratory insufficiency due to flail chest, thoracic wall deformity, and sternal dislocation (*n* = 1), two sternal fractures in combination with multiple bilateral rib fractures (*n* = 1), or for general biomechanical support (*n* = 11). The latter was defined by Krinner [3] as a surgical indication formulated by Harston [30]; however, it was not further specified which indication applied to which patient.

50 patients (51%) received conservative treatment for their spinal fractures, consisting of haloframe (*n* = 7, 14%) or other treatment (*n* = 42, 86%), such as a Philadelphia collar or no additional treatment. 48 patients (49%) underwent spinal surgery. Of the 49 patients with unstable spinal fractures, 42 (86%) were surgically treated and 3 patients were treated with haloframe because of subaxial cervical fractures. Four patients were treated conservatively despite unstable spinal fractures: one patient had a benign neglect policy due to old age, one had severe psychiatric disease upon hospital admission, one had a unilateral facet fracture, and one patient could not afford spinal surgery. One patient with a neurological deficit was treated conservatively, because of a spinal cord lesion in the presence of a stable type A spinal fracture (Table 5).

**Table 5:** Treatment methods and outcomes^a^

Sternal fractures	Overall *n* = 98		Sternum CONS Spine CONS *n* = 43		Sternum CONS Spine OP *n* = 41		Sternum OP Spine CONS *n* = 7		Sternum OP Spine OP *n* = 7		*p* value
Primary sternal treatment, *n* (%)											
Conservative	84	(86)									
Operative	14	(14)									
Sternal treatment failure, *n* (%)	5	(5)	3	(7)	2	(5)	0		0		1.000
Secondary operation	2	(2)	1	(2)	1	(2)					
Surgical indication	1	(1)	0		1	(2)					
Unknown treatment outcome	2	(2)	2	(5)	0						
Spinal fractures											
Primary spinal treatment, *n* (%)											
Conservative	50	(51)									
Operative	48	(49)									
Conservative treatment method, *n* (%)											
Haloframe	7	(14)	6	(14)			1	(17)			
Other	42	(86)	37	(86)			5	(83)			
Spinal treatment failure, *n* (%)											
Overall^b^	11	(11)	5	(12)	6	(15)	0		0		0.786
Biomechanical failure	8	(8)	5	(12)	3	(7)	0		0		0.921
Reoperation	2	(2)			2	(5)			0		
Secondary operation	4	(4)	3	(7)	1	(2) ^f^	0				
Treatment refusal	2	(2)	2	(5)	0						
Other treatment outcomes											
Pneumonia, *n* (%)^c^	29	(30)	14	(33)	15	(37)	0		0		0.068
Wound infection, *n* (%) ^c,d^	6	(10)	1	(2)	5	(12)	0		0		0.602
Hospital LOS in days, median [IQR]^e^	17	[8–24]	12	[5–25]	17	[8–23]	24	[17–24]	24	[24–24]	0.072

*CONS* conservative treatment, *OP* operative treatment, *LOS* length of stay, *IQR* interquartile range

^*^Statistically significant difference (*p* < 0.05)

^a^Due to rounding off, percentages might not add up to 100%

^b^Overall treatment failure included technical failure (malpositioned screws and pain due to osteosynthesis materials)

^c^Only reported by Klei et al. [24]

^d^Wound infection in conservative treatment group was caused by a haloframe pintract infection

^e^Not reported by Labbe et al. [10], Regauer et al. [26], and Jiang et al. [11]

^f^This patient was included in the operative spinal treatment group due to his thoracic fracture, but secondary dislocation of a conservatively treated odontoid fracture occurred

### Treatment outcomes

Sternal treatment failure occurred in five patients (5%). Of these patients, three were treated conservatively for both their sternal and spinal fracture (SternumCONS/SpineCONS group). One patient underwent secondary operation due to dislocation of a sternal body fracture, in combination with increasing kyphosis of the thoracic spine (for which he refused treatment); two patients had unknown treatment outcomes for their sternal fracture. The other two patients with sternal treatment failure were included in the SternumCONS/SpineOP group. One patient underwent secondary operation due to secondary sternal dislocation. One patient had a missed fracture-dislocation of the manubriosternal joint, which subsequently showed secondary dislocation and non-union (Tables 5 and 6).

**Table 6:** Treatment failure

	Sternal fracture	Spinal fracture	Treatment group	Spinal treatment indication	Sternal treatment failure	Spinal treatment failure
1	MS-joint	T3-4:A;N0	SternumCONS/SpineCONS	Stable	Unknown outcome	Treatment refusal (increasing kyphosis)
2	Body with dislocation	T4-8:B2;N0	SternumCONS/SpineCONS	Unstable (treatment refusal)	SecOP (secondary dislocation)	Treatment refusal (increasing kyphosis)
3	Body	T3:A1;T5:A1;N0	SternumCONS / SpineCONS	Stable	Unknown outcome	–
4	Body	C4-C5:B2;N0	SternumCONS / SpineCONS	Unstable (haloframe)	–	SecOP (secondary dislocation)
5	Manubrium	C2:A(dens);T9:A1;N0	SternumCONS / SpineCONS	Stable	–	SecOP (secondary dislocation of dens)
6	Manubrium and body	T3-T6:B2;T4:A1;T5:A3;N0	SternumCONS / SpineCONS	Unstable (psychiatric illness)	–	SecOP (secondary dislocation)
7	Body	T3-T5:B2;T4:A2;N0	SternumCONS / SpineOP	Unstable	SecOP (secondary dislocation)	ReOP (secondary dislocation)
8	MS-joint and body	L2:B1;N0	SternumCONS / SpineOP	Unstable	SurgInd (secondary dislocation and NU)	–
9	Body	T6-T7:B2;T7:A2;N0	SternumCONS / SpineOP	Unstable	–	ReOP (non-union)
10	Manubrium	C2:A;T4-T6:B2;T4:A1;T5:A3;N3	SternumCONS / SpineOP	Unstable	–	SecOP (secondary dislocation of dens)

*MS-joint* manubriosternal joint, *CONS* conservative treatment, *OP* operative treatment, *SecOP* secondary operation, *SurgInd* surgical indication, *ReOP* re-operation, *NU* non-union

Overall, 11 patients (11%) displayed spinal treatment failure. Biomechanical spinal treatment failure occurred in eight patients (8%). Technical failure occurred in three patients who underwent re-operation due to malpositioned screws (*n* = 2) and severe pain from pedicle screws (*n* = 1). Five of the eight patients with biomechanical spinal failure were included in the SternumCONS/SpineCONS group. Three patients had a secondary operation due to secondary dislocation; however, in one of these patients this secondary dislocation occurred in an associated odontoid fracture. Two patients refused treatment despite increasing spinal kyphosis with a surgical indication. Based on the AOSpine surgical algorithm, one patient who refused treatment and one patient with secondary dislocation of a conservatively treated unstable spinal fracture, should have undergone primary surgical treatment due to unstable fractures [31]. The other three patients with biomechanical failure were part of the SternumCONS/SpineOP group. Two had secondary dislocation resulting in reoperation and secondary operation, respectively. One patient was surgically treated for his unstable thoracic spinal fractures, but showed secondary dislocation of a conservatively treated odontoid fracture which resulted in secondary operation. Notably, three patients displayed both sternal and spinal treatment failure (Tables 5 and 6).

29 patients (30%) developed a pneumonia during their hospital stay. Six patients (10%) had a wound infection, of whom one patient had a haloframe pin tract infection (pneumonia and wound infection were only reported by Klei [2]). Median hospital length of stay was 17 days (IQR 8–24 days), but was not reported by Labbe [10], Regauer [26], and Jiang [11].

There were no significant differences whatsoever in treatment outcomes between the treatment groups (Table 5).

## Discussion

In the present systematic review of sternovertebral fracture treatment, five patient (5%) showed sternal treatment failure and eight patients (8%) displayed biomechanical spinal treatment failure. Only 14 patients (14%) underwent sternal fixation; the majority of patients received conservative sternal treatment. Notably, sternal treatment failure only occurred after conservative sternal treatment. However, sternal treatment failure was perhaps overestimated, since two patients with unknown treatment outcomes were counted as treatment failure, and one patient had a missed fracture that should have been treated surgically. Moreover, most indications for sternal fixation could not be verified and some might have been superfluous.

Spinal treatment failure was likely overestimated as well. Of the eight patients with biomechanical spinal failure, two showed secondary dislocation of an associated odontoid fracture, which might not biomechanically be influenced by a sternal fracture. Spinal treatment strategies were largely based on the presence of unstable spinal fractures and neurological deficits. In retrospect, based on the AOSpine surgical algorithm, two patients with spinal failure should have undergone primary operative treatment for unstable spinal fractures; however, one patient refused treatment and in one patient, stability of the spinal fracture was likely misinterpreted.

There were no significant differences in sternal and spinal treatment outcomes between the treatment groups. The findings of this systematic review therefore suggest that sternal fixation is not imperative to achieve sternal and spinal healing, provided that the generally accepted indications for sternal fixation [30] and the AOSpine surgical algorithm for unstable spinal fractures are followed [31].

Sternovertebral fractures are rare injuries [1, 2, 15, 22]. Few studies have been published on these injuries and their treatment. In a period of almost 30 years, 6 studies were published with mostly few patients; the largest study was a cohort study conducted at our level-1 trauma centre and included 73 patients. To date, no systematic review has been conducted on this topic.

The majority of sternal injuries were located at the sternal body (55%) or the manubrium (43%). Ten patients (10%) showed a sternal dislocation. Although dislocation is considered an indication for sternal fixation [25], seven of these patients received conservative sternal treatment. Only one of them showed sternal treatment failure, perhaps suggesting that indications for sternal fixation [30] should be revised.

The majority of patients had spinal fractures of the thoracic and thoracolumbar spine (96%), due to the fact that all studies excluded patients with isolated cervical and lower lumbar fractures. Although the association between sternal and thoracic spinal fractures is widely assumed in literature [1, 4, 7–9, 12], the distribution of spinal fractures over the different spinal regions has not been established unambiguously [1, 2, 4–6, 13–20]. For instance, in our own cohort study, 14 out of 87 sternovertebral fracture patients (16%) had only upper cervical and/or lower lumbar fractures [2]. The location and severity of spinal fractures might depend on the location of the corresponding sternal fracture [5, 32].

This systematic review was based on six studies, five of which had a low quality score. Although the applicability of MINORS criteria to case reports might be limited, case reports and case series are known for potential selection and reporting bias, which was reflected in the quality scores. Studies had a heterogeneous study population and different treatment strategies: for instance, all sternal fractures described by Labbe [10] were conservatively managed, while sternal fractures reported by Krinner [3] were surgically treated. The latter study only reported treatment outcomes for 11 out of 32 patients who underwent sternal fixation, reflecting the risk of reporting bias. Some treatment groups were therefore highly skewed by a single study. The diverse treatment choices, without clear surgical indications, reflect a lack of evidence and standardised treatment guidelines. Moreover, the occurrence of pneumonia and wound infection was only reported in our own cohort study and therefore limited to the SternumCONS/SpineCONS and SternumCONS/SpineOP treatment groups [2].

In conclusion, limited studies have been published on the treatment of sternovertebral fractures and randomised trials are lacking. Six studies were included in the current review, with a total of 98 patients. Most studies were of low quality and had heterogeneous treatment strategies. Therefore, results should be interpreted with caution. Despite these limitations, treatment outcomes did not significantly differ between treatment groups. These findings indicate that for most patients with sternovertebral fractures, conservative sternal treatment is safe and effective. Sternal fixation is not essential to achieve sternovertebral stability provided that spinal fractures are treated according to the AOSpine surgical algorithm.

## Electronic supplementary material

Below is the link to the electronic supplementary material.

Supplementary file1 (DOCX 24 kb)

## Data Availability

Not applicable.
