# Supplementary material for: Current treatment and outcomes of traumatic sternovertebral fractures: a systematic review
Source: Eur J Trauma Emerg Surg. 2020 Oct 1;47(4):991–1001. doi: 10.1007/s00068-020-01505-y (PMC8322016; doi:10.1007/s00068-020-01505-y)
Supplement: Supplementary file 1 — Supplementary file1 (DOCX 24 kb) [file 68_2020_1505_MOESM1_ESM.docx]

# APPENDIX A – LITERATURE SEARCH

PubMed

((((("Sternum"[Mesh] OR Sternum[Title/Abstract] OR Sternal[Title/Abstract] OR Manubrium[Title/Abstract] OR Xiphoid[Title/Abstract] OR Breastbone[Title/Abstract])) AND ("Spine"[Mesh] OR Spine[Title/Abstract] OR Spinal[Title/Abstract] OR Vertebr*[Title/Abstract] OR Column[Title/Abstract] OR Cervical[Title/Abstract] OR Thoracic[Title/Abstract] OR Thoracolumbar[Title/Abstract] OR Lumbar[Title/Abstract] OR Corpus[Title/Abstract] OR Body[Title/Abstract] OR “Spinous process”[Title/Abstract] OR “Transverse process”[Title/Abstract] OR Arch[Title/Abstract] OR Atlas[Title/Abstract] OR Axis[Title/Abstract] OR Dens[Title/Abstract])) AND (("Fractures, Bone"[Mesh] OR Fracture*[Title/Abstract] OR Broken[Title/Abstract]) OR ("Joint Dislocations"[Mesh] OR Dislocation*[Title/Abstract] OR Diastasis[Title/Abstract] OR Luxation*[Title/Abstract] OR Subluxation*[Title/Abstract]))) AND (("Wounds and Injuries"[Mesh] OR Wound*[Title/Abstract] OR Injur*[Title/Abstract] OR Trauma*[Title/Abstract]) NOT ("Osteoporosis"[Mesh] OR Osteoporos*[Title/Abstract] OR Decalcification*[Title/Abstract] OR (Bone[Title/Abstract] AND Loss*[Title/Abstract])))) AND ("therapy" [Subheading] OR Therap*[Title/Abstract] OR Treat*[Title/Abstract] OR Intervention*[Title/Abstract] OR Conservative[Title/Abstract] OR Surger*[Title/Abstract] OR Surgical[Title/Abstract] OR Operation*[Title/Abstract] OR Operative[Title/Abstract] OR Manage*[Title/Abstract])

Embase/MEDLINE

'sternum'/exp OR 'sternum':ti,ab OR 'sternal':ti,ab OR 'manubrium':ti,ab OR 'sternal body':ti,ab OR 'corpus sterni':ti,ab OR 'xiphoid':ti,ab OR 'processus xiphoideus':ti,ab OR 'breastbone':ti,ab AND ('spine'/exp OR 'spine':ti,ab OR ‘spinal’:ti,ab OR vertebr*:ti,ab OR ‘column’:ti,ab OR ‘cervical’:ti,ab OR ‘thoracic’:ti,ab OR ‘thoracolumbar’:ti,ab OR ‘lumbar’:ti,ab OR ‘corpus’:ti,ab OR ‘body’:ti,ab OR ‘spinous process’:ti,ab OR ‘transverse process’:ti,ab OR ‘arch’:ti,ab OR ‘atlas’:ti,ab OR ‘axis’:ti,ab OR ‘dens’:ti,ab) AND ('fracture'/exp OR 'fractur*':ti,ab OR ‘broken’:ti,ab OR ('dislocation'/exp OR ‘diastasis’:ti,ab OR ‘luxation*’:ti,ab OR ‘subluxation*’:ti,ab)) AND ('injury'/exp OR 'injur*':ti,ab OR 'trauma*':ti,ab OR 'wound*':ti,ab NOT ('osteoporosis'/exp OR 'osteoporo*':ti,ab OR 'decalcification':ti,ab OR ('bone':ti,ab AND ('loss':ti,ab OR 'losses':ti,ab)))) AND ('therapy'/exp OR ‘therap*’:ti,ab OR ‘treat*’:ti,ab OR ‘intervention*’:ti,ab OR ‘conservative’:ti,ab OR ‘surger*’:ti,ab OR ‘surgical’:ti,ab OR ‘operation*’:ti,ab OR ‘operative’:ti,ab OR ‘manage*’:ti,ab)
